# Probing the Effect of Ultrasonication, Probiotic Lacto‐Fermentation, and Blanching on Bioactive Compounds, Antioxidants Activities, and Antinutrients of Tomato

**DOI:** 10.1002/fsn3.70970

**Published:** 2025-09-14

**Authors:** Abdul Rehman Ayub, Muhammad Waseem, Zulfiqar Ahmad, Jaza Maqbl Alshammari, Tariq Ismail, Muhammad Ammar Khan, Muhammad Rizwan Javed, Muhammad Saleem, Rana Muhammad Aadil, Muhammad Faisal Manzoor, Muhammed Adem Abdullahi

**Affiliations:** ^1^ Department of Food Science and Technology, Faculty of Agriculture and Environment The Islamia University of Bahawalpur Bahawalpur Pakistan; ^2^ Faculty of Biological and Agriculture Engineering, Applied College Al‐Baha University Al‐Baha Saudi Arabia; ^3^ Department of Food Science and Technology, Faculty of Food Science and Nutrition Bahauddin Zakariya University Multan Multan Pakistan; ^4^ National Institute of Food Science and Technology University of Agriculture Faisalabad Pakistan; ^5^ Guangdong Provincial Key Laboratory of Intelligent Food Manufacturing, School of Food Science and Engineering Foshan University Foshan China; ^6^ Faculty of Sciences and Technology ILMA University Karachi Pakistan; ^7^ Department of Food Science and Postharvest Technology, Jimma University College of Agriculture and Veterinary Medicine Jimma University Jimma Ethiopia

**Keywords:** fermentation, *Lycopersicum esculentum*, micronutrients, processing, tomatoes, tubers

## Abstract

Present study was conducted to assess the lactic acid fermentation (LAB, 24 h), ultrasonication (10, 20, and 30 min), and blanching (90°C, 1 min) on the nutrients, antinutrients, and antioxidants. LAB fermentation reported the presence of *L. plantarum* and total plate counts of 4.10 and 1.02 log_10_ CFU/g, respectively. However, biological, thermal, and non‐thermal processed tomato powder exhibited the highest ash, fibers, proteins, Na, K, Ca, Mg, and Fe, that is, 11.82 g/100 g, 8.22 g/100 g, 22.35 g/100 g, 792 mg/100 g, 2246 mg/100 g, 159 mg/100 g, 271 mg/100 g, and 5.6 mg/100 g, respectively. Among various treatments, LAB fermentation anticipated the highest decline in phytates, oxalates, glycoalkaloids, and saponins by 95%, 94%, 87%, and 89%, respectively. Similarly, LAB fermentation resulted in the maximum contents of lycopene, β‐carotene, and ascorbic acid, that is, 177 mg/100 g, 14.7 mg/100 g, and 113 mg/100 g, respectively. Likewise, the maximum total phenolic contents (TPC), total flavonoid contents (TFC), and 2,2‐diphenyl‐1‐picrylhydrazyl (DPPH) were measured in the fermented tomato powder, that is, 765 mg GAE/100 g, 647 mg QE/100 g, and 87%, respectively. The findings suggest that LAB fermentation is the most effective treatment for reducing the antinutrients by contributing to the safety profile of tomatoes and increasing health‐promoting nutrients and antioxidants.

## Graphical abstract

The present study was investigated to assess the effect of lactic acid fermentation (LAB, 24 h), ultrasonication (10, 20, and 30 min), and blanching (90°C, 1 min) on the nutrients, antinutrients, and antioxidant activities. Among various processing treatments, LAB fermentation anticipated the highest decline in phytates, oxalates, glycoalkaloids, and saponins by 95%, 94%, 87%, and 89%, respectively. The findings suggest that LAB fermentation is the most effective treatment for reducing the antinutrients by contributing to the safety profile of tomatoes and increasing health‐promoting nutrients and antioxidants.

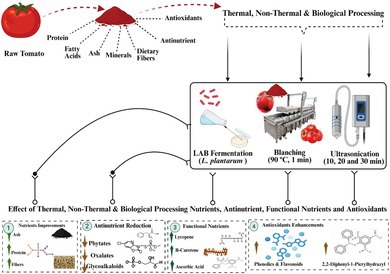

## 1 Introduction

Tomato ( *Solanum lycopersicum* L.), a vital vegetable food crop that belongs to the family Solanaceae (Taha et al. 2023); China, India, Turkey, and Italy are among the leading producers (Caruso et al. 2024). According to the World Tomato Processing Council report, more than 130 million tons of tomatoes are processed worldwide each year (Amrith et al. 2024). Tomatoes are enriched sources of dietary fibers, vitamins (A, E, K, and B), minerals (Zn, Fe, K, Na, and Mg), phenolics (phenolic acids, caffeic acid, chlorogenic acid, ferulic acid, gallic acid, and protocatechuic acid), flavonoids (rutin naringenin, kaempferol chalcone, and quercetin), and carotenoids (β‐carotene, α‐carotene, phytoene, phytofluene, lutein, zeathanxin, and neurosporene) (Chabi et al. 2024; Wang et al. 2024; Wu et al. 2022). These biologically active compounds exhibit several health‐promoting activities against disorders such as oxidative stress, metabolic dysfunctions, inflammation, obesity, and diabetes mellitus (Boulaajine and Hajjaj 2024; Echresh et al. 2024; Shafe et al. 2024).

Despite the high nutraceutical potential of tomatoes, the tuber plant also contains a sufficient magnitude of antinutrients such as oxalates, phytates, and saponins (Shuaibu 2022). These antinutrients are recognized to interfere with the metabolic fate of proteins and minerals, thereby decreasing their bioavailability (Rahmati‐Joneidabad et al. 2024; Waseem et al. 2024). Phytates, oxalates, and saponins are known to pose adverse health effects in humans, such as kidney stones (nephrolithiasis), hemolysis, paralysis, antienzyme activities, hyperactivity, and even death (Eleazu et al. 2020). Long‐term consumption of antinutrients may lead to impaired intestinal integrity, lower iron, and calcium (hyposideremia and hypocalcemia) in blood plasma, and several immunological disorders (Arsov et al. 2024).

In this connection, earlier studies have shown notable potential in various processing techniques like thermal (autoclaving and boiling), non‐thermal (germination, soaking, and extrusion), and biological (fermentation) on the reduction of these toxic antinutrients in fruits and vegetables (Waseem et al. 2024). Comparatively, conventional processing techniques may not guarantee achieving the safer limits of antinutrients like oxalates. Therefore, biological techniques like fermentation and non‐thermal techniques like ultrasonication have gained popularity among the research community owing to their better efficiencies in reducing the ever‐increasing burden of antinutrients (Kahala et al. 2021; Tahir et al. 2023). In particular, in biological processing, LAB fermentation has been reported as a promising method for mitigating antinutrients such as oxalates and phytates (Behbahani, Noshad, et al. 2024; Behbahani et al. 2023; Layla et al. 2024). LAB are typically the dominant probiotics in fermented vegetables, and *Lactiplantibacillus plantarum* has already been used commonly as a probiotic due to its appreciable health‐promoting features by decreasing the growth of spoilage bacteria and maintaining gut health (Behbahani and Noshad 2024; Echegaray et al. 2023; Yuan et al. 2023).

Presence of antinutrients in tomatoes above safe limits resulting in losses to human health. Likewise, the use of traditional methods in mitigating the toxicants associated with tomatoes has shown limited success, with no significant progress which indicates a clear research gap. Therefore, this research was aimed at investigating the potential of fermentation, blanching, and ultrasonication as viable thermal, non‐thermal, and biological techniques to mitigate toxicogenic antinutrients to enhance nutritional, antioxidant, safety, and food value addition properties of tomatoes for commercial applicability and safer quality.

## 2 Materials and Methods

### 2.1 Procurement of Raw Materials, Reagents, and Chemicals

Fresh tomatoes (~10 kg) were procured from the local Imtiaz Super Market, Bahawalpur, Punjab, Pakistan. All chemicals and reagents used in the study, including Folin–Ciocalteu reagent, Folin‐Denis reagent, sulfuric acid (H_2_SO_4_), acetone, methanol, sodium carbonate (Na_2_CO_3_), magnesium carbonate (MgCO_3_), meta‐phosphoric acid, ammonium iron (III) sulfate solution, sodium acetate, potassium acetate, ammonium hydroxide, ferrous sulfate, and aluminum chloride were purchased from Sigma‐Aldrich Co. Ltd. (Steinheim, Germany) and Merck (Darmstadt, Germany). *Lp. plantarum* (ATCC 8014) strains for fermentation were procured from Fengchen Group Co., China.

### 2.2 Biological, Thermal and Non‐Thermal Processing of Tomatoes

#### 2.2.1 Fermentation

At first, *Lp. plantarum* culture was activated using DeMan‐Rogosa‐Sharpe (MRS) broth by incubating at 37°C for 48 h. After the incubation period, freshly produced bacterial cell biomass was separated by centrifugation (Hermle Z236K, Wehingen, Germany) at 6000 *g* for 10 min and suspended in 0.9% saline water. Then, *Lp. plantarum* cells recovered as sediments in the falcon tube were washed by re‐suspending in normal sterile saline to obtain a final cell count of 10^−6^ CFU/mL. Thereafter, tomato samples kept for fermentation were immersed in sterile water already inoculated with the freshly prepared *Lp. plantarum* culture at 10^−6^ CFU/g. The fermentation jar was incubated at 37°C for 120 h (Naseem et al. 2023). Subsequently, the microbial enumeration was performed by total plate count (TPC) method using the DeMan‐Rogosa‐Sharpe (MRS) agar followed by incubation at 37°C for 48 h, and colonies were enumerated using a colony counter (Model CC‐J2; Infitek Co. Ltd., Shandong, China). The results were expressed as CFU/g.

#### 2.2.2 Blanching

Consistently, even or graded fresh tomatoes were blanched in the hot water (1:10 w/v) at 90°C ± 2°C for 1 min. The extraneous surface water was drained off, and blanched tomatoes were pre‐dried using a clean muslin cloth.

#### 2.2.3 Ultrasonication

Tomatoes were ultrasonicated in an ultrasonic bath cleaner (SKYMEN JP‐031S, Shenzhen, China) at a power of 180 W radiation, a frequency of 40 kHz, and a time of 10, 20, and 30 min at 30°C (Manzoor et al. 2021). Untreated and treated tomato samples were spread evenly over the nylon trays (mesh size = 0.186 × 0.186 m^2^) and dehydrated at 45°C for 10 h using a cabinet dryer (Pamico Tech, Faisalabad, Punjab, Pakistan) to maintain the 8%–10% moisture level. Subsequently, dehydrated tomatoes were converted into fine powder (mesh size ~72 mm) using a heavy‐duty grinder (Waseem et al. 2024).

### 2.3 Determination of pH and Titratable Acidity

pH of all the samples of tomatoes was measured by adopting the method of Yuan et al. (2023) using a pH meter (PB‐10; Sartorius, Trading Co. Ltd., Shanghai, China). Using the titrimetric method, titratable acidity of all tomato samples was measured following the method of Li et al. (2025). For this, 10 g of each sample was accurately measured in 10 mL distilled water and 0.1 N NaOH using phenolphthalein as an indicator. The emergence of a light pinkish endpoint exhibited the completion of titration. The results for the titratable acidity were expressed as %.

### 2.4 Nutritional Composition

The nutritional composition of untreated and treated tomato samples was performed by adopting the standard protocols as documented in the Association of Official Analytical Chemists (AOAC), that is, moisture (method no. 925.10), ash (method no. 923.03), fat (method no. 920.85), fiber (method no. 32‐10), and protein (method no. 920.87), respectively. Carbohydrate contents were calculated by using the following equations:

(1) $$\text{Carbohydrates}\;\left(g/100\;g\right)=\left[100-\left(\text{moisture}+\mathrm{ash}+\mathrm{fat}+\text{fiber}+\text{protein}\right)\right]$$

Mineral contents of untreated and treated tomato samples were determined per the AOAC guidelines (Latimer 2019).

### 2.5 Chlorophyll *a*, *b*, and Total Chlorophyll Determination

Precisely measured 1.0 g of each tomato powder sample was mixed in an accurate volume of 10 mL, 80% acetone, and 0.5 g of magnesium carbonate. The sample admixture was placed in a refrigerator for 4 h. Afterward, the mixture was centrifuged (Hermle Z236K, Wehingen, Germany) at 5000 rpm for 5 min. Subsequently, the spectrophotometric (UV‐3000; VWR, Darmstadt, Germany) absorbances of each sample and reagent blank (i.e., 80% acetone) were recorded at 645 and 663 nm, respectively (Younis et al. 2024). The total chlorophyll contents were quantified by following the equations:

(2) Chlorophylla=11.75×Absorbanceat663−2.35×Absorbanceat645

(3) Chlorophyllb=18.61×Absorbanceat645−3.96×Absorbanceat663

(4) Total chlorophyll=Chlorophylla+chlorophyllb

### 2.6 Lycopene Determination

Lycopene of untreated and treated tomato samples was performed by adopting the protocol Rani and Vijayanchali (2024). Accurately measured 1 mg of each sample was mixed in accurately measured 8 mL of acetone:ethanol:hexane at 1:1:2 ratios. Thereafter, 1 mL of distilled water was also added, and the mixture was allowed to react for 10 min for the emergence of a bright light color on phase separation. Lycopene contents of each sample against the reagent blank were recorded at 503.

(5) $$\text{Lycopene}\;\left(\mathrm{mg}/100\;g\right)=A503\times 17.17\times \text{Volume of mixed solvent}$$

### 2.7 β‐Carotene Determination

The beta‐carotene contents of untreated and treated tomato samples were estimated by adopting the method outlined by Soytong et al. (2021). About 500 mg of each tomato sample was accurately measured twice with the chilled acetone and allowed to stand in an ice bath for 15 min. Then, the mixture was vortexed vigorously for 10 min and centrifuged (Hermle Z236K, Wehingen, Germany) at 1370 *g* for 10 min. Following centrifugation (Hermle Z236K, Wehingen, Germany), the supernatants of each sample were filtered using the Whatman filter no. 42. Finally, the spectrophotometric absorbances of each supernatant from the samples were measured at 449 nm against the standard curve plotted for the beta‐carotene standard for its concentration measurement, and mean values were estimated as mg/100 g.

### 2.8 Ascorbic Acid Determination

The ascorbic acid content of tomato samples was measured using the protocols of Ismail et al. (2024). Accurately measured 0.5 g of each tomato sample was mixed in 50 mL of 5% *meta*‐phosphoric acid and 10% acetic acid solution in a conical flask. The reaction mixture of each sample was then poured into a 100 mL volumetric flask and mixed for homogeneity. The volume was adjusted using the 5% *meta*‐phosphoric acid and 10% acetic acid solutions. Following that, the resulting solution mixture was filtered. A few drops of bromine water were added to the filtrate to oxidize ascorbic acid. Afterwards, some drops of 10% thiourea were poured into the filtrate mixture to remove surplus bromine residues. Then, 1 mL of 2,4 dinitrophenylhydrazine (DNPH) solution and ascorbic acid standards (5–25 μg/mL) were accurately measured. Samples along with the standards were incubated at 37°C for 3 h, cooled in an ice bath, and 5 mL of concentrated sulfuric acid was added to it. Spectrophotometric absorbances of each sample and the calibration curve standards were taken at 521 nm.

### 2.9 Antinutrients Determination

#### 2.9.1 Phytates

Phytates of all tomato samples were estimated using the following methodology (Waseem et al. 2022). One gram of each sample was mixed in 10 mL of 0.2 N hydrochloric acid with constant stirring for 30 min. Then, 0.5 mL of the extract was taken and poured into 1 mL of a ferric solution containing ammonium iron (III) sulfate solution. Thereafter, the reaction mixture was subjected to boiling for 30 min, allowed to rest for 30 min, and centrifuged (Hermle Z236K, Wehingen, Germany) at 3000 rpm for 30 min. Following this, 1 mL of the centrifuged supernatant was shifted to the Erlenmeyer flask already containing 1.5 mL of 2,2′‐bipyridine solution (i.e., 0.25 g each of thioglycolic acid and 2,2′‐bipyridine were mixed in distilled water and the final volume was adjusted to 25 mL). Spectrophotometric absorbance of all samples and the reagent blank was recorded at 519 nm against phytate‐phosphorous (100–1000 mg/L) standards. Phytate contents were calculated against phytic acid standard curves using distilled water as a reagent blank.

#### 2.9.2 Oxalates

The oxalates contents of all tomato samples were estimated as documented by Michel et al. (2023). Each sample's known quantity of 2.0 g was mixed in 200 mL of deionized water, followed by 20 mL of 6 N hydrochloric acid. Thereafter, the reaction mixture was heated for 1 h and filtered using Whatman filter paper no. 41. About 50 mL of the filtrate was again homogenized in 20 mL of 6 N hydrochloric acid and filtered. Subsequently, the 50 mL of the filtrate was subjected to mixing in methyl red indicator (0.1%, w/v), concentrated ammonia, heated for a while, and filtered again. Afterward, the filtrate was boiled and mixed in 5% calcium chloride (CaCl_2_) for the development of crystals of calcium oxalates and filtered again. Thereafter, filtration residues were washed using boiling distilled water, subjected to an oven (Memmert UNB 200, Munich, Germany), and mixed in 10 mL of diluted sulfuric acid. Titration was performed against 0.05 N potassium permanganate (KMnO_4_) solutions. Oxalates were calculated as mg/100 g.

#### 2.9.3 Alkaloids

Tomatoes were estimated for alkaloids by following the protocol as adopted by Naseem et al. (2023). About 5.0 g of each sample was poured into 50 mL of 10% acetic acid solution in ethanol. Then, the reaction admixture was gently shaken and rested for 4 h at room temperature. Thereafter, the filtration was performed using filter paper no. 41, and the filtrate was subjected to evaporation to obtain one‐quarter of the actual volume. Subsequently, concentrated ammonium hydroxide (NH_4_OH) was poured gently to obtain alkaloid precipitates. Finally, the alkaloid precipitates were filtered using filter paper no. 41 and weighed along with the filter paper. Final washing of the alkaloid precipitates was performed using 1% ammonium hydroxide solution, and alkaloid contents in untreated and treated tomato samples were calculated as mg/100 g.

#### 2.9.4 Saponins

A study by Perveen et al. (2024) was followed to estimate saponins in tomato samples. About 0.25 g of each sample was diluted with 0.25 mL of 8% vanillin solution (i.e., already developed in the ethanol) and 72% 2.5 mL sulfuric acid. Then, the prepared sample admixture, reagent blank (solution without sample), and standard (aescin) were allowed to react at 60°C for 15 min. After the reaction, the admixtures were cooled for 5 min at room temperature. Spectrophotometric absorbances of each sample, reagent blank, and standards (0–100 ppm) were recorded at 560 nm. Saponin contents were estimated as mg/100 g.

### 2.10 Antioxidants Determination

#### 2.10.1 Total Phenolic Content (TPC)

TPC of untreated and treated tomato samples was determined by following the methodology mentioned by Liang et al. (2023). Precisely, 0.3 mL extract was mixed in 2.5 mL, 0.2 N Folin–Ciocalteu reagent (FCR). Thereafter, the mixture was incubated for 5 min at room temperature, followed by the addition of saturated sodium carbonate solution (i.e., 75 g/L). Then, the admixture was incubated at room temperature for 1 h. Spectrophotometric absorbances were measured at 765 nm against the reagent blank and gallic acid standards (50–500 ppm). TPC mean values were expressed as mg of gallic acid equivalents per gram.

#### 2.10.2 Total Flavonoid Contents (TFC)

For TFC determination, a protocol as documented by Quyen et al. (2020) was followed. Accurately measured, 0.5 mL of each sample was mixed in 0.1 mL of 10% aluminum chloride, 0.1 mL of 1 M potassium acetate, and 4.3 mL distilled water. Thereafter, the reaction mixture was incubated at room temperature for 30 min. Spectrophotometric absorbances of each sample, reagent blank, and quercetin standards (10–1000 ppm) were measured at 415 nm. TFC mean concentrations were expressed as μg of quercetin equivalents per gram.

#### 2.10.3 2,2‐Diphenyl‐1‐Picrylhydrazyl (DPPH)

All tomato samples were estimated for the determination of DPPH assay by adopting the methodology of Koçak (2024). One millimeter of 0.1 mM DPPH solution was poured into an accurately measured sample extract of 3 mL. The resulting mixture was mixed with constant stirring and incubated for 30 min. Subsequently, spectrophotometric absorbance of each sample, reagent blank, and standards was taken at 517 nm. The DPPH free‐radical scavenging activities (%) were calculated using the following equation

(6) $$\text{DPPH}\;\left(\%\right)=\frac{\text{Absorbance of control}-\text{Absorbance of sample}}{\text{Absorbance of control}}\times 100$$

#### 2.10.4 Ferric Reducing Antioxidant Power (FRAP)

FRAP activities of untreated and treated tomato samples were determined by adopting the methodology outlined by Bratovcic et al. (2021). FRAP reagent was prepared by dissolving about 200 mL of sodium acetate buffer solution (300 mmol/L, pH 3.6), 20 mL of tripyridyl triazine (TPTZ) solution (conc. 10 mmol/L in 40 mmol/L HCl), 20 mL of ferric chloride solution (conc. 20 mmol/L), and 24 mL of distilled water. About 0.2 mL of methanolic extract of each sample and about 3.8 mL of FRAP reagent were mixed and incubated at 25°C for 4 min. Spectrophotometric absorbance of each sample, reagent blank, and ferrous sulfate standards (conc. ~10–1000 ppm) was estimated at 593 nm. FRAP values of all samples were measured in mmol FeSO_4_ equivalents, and findings were expressed in mg/100 g.

### 2.11 Statistical Analysis

All experiments, including nutritional composition, mineral composition, antioxidants, and antinutrients, were performed in duplicates as two independent experiments, and mean values were expressed as ±standard deviation (SD). These results were subjected to the analysis of variance (ANOVA) technique, and the least significant difference (LSD) test was used to find a level of significance (*p* < 0.05) at a 5% confidence interval using the Statistix 8.1 (Tallahassee, FL, USA).

## 3 Results and Discussion

### 3.1 Nutritional Composition of Blanched, Fermented and Ultrasound‐Treated Tomato Powder

**FIGURE 1:**
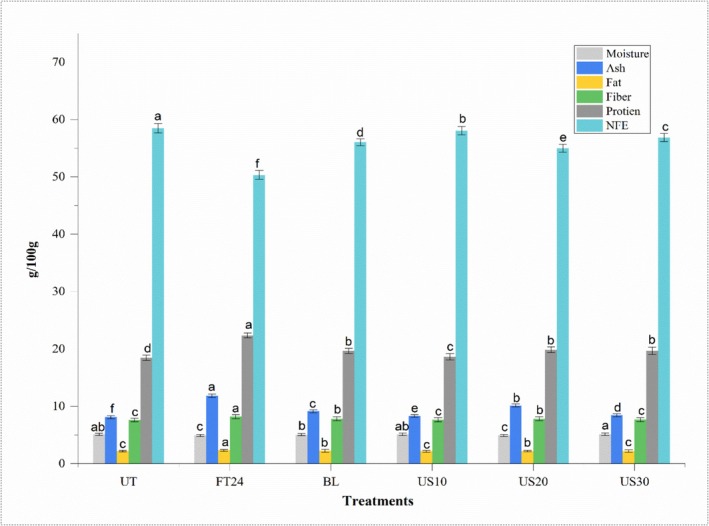
Nutritional composition of untreated tomatoes (UT), fermentation 24 h (FT24), blanching (BL), ultrasonication 10 min (US10), ultrasonication 20 min (US20), and ultrasonication 30 min (US30). Values are expressed as means ± SD (*n* = 2). Mean values presenting similar lettering are statistically nonsignificant (*p* > 0.05). NFE, nitrogen‐free extract (i.e., carbohydrates).

Results for the nutritional composition of untreated and treated tomato powders revealed the LAB fermentation to exhibit the highest ash, proteins, and fibers, that is, 11.8, 24.4, and 8.2 g/100 g, respectively, followed by the lowest mean values in control, that is, 8.1, 18.5, and 7.6 g/100 g, respectively (Figure 1). Also, among the ultrasonication treatments, ultrasonication at 20 min elucidated the highest ash, protein, and fiber contents, that is, 10, 7.8, and 19.8 mg/100 g, respectively. Our findings suggest that LAB fermentation enhanced the nutritional value of tomato powder to the highest level. The increase in protein contents of tomato powder during fermentation could be linked with the increase in bacterial biomass and transformation of inorganic nitrogen to organic nitrogen. The increase in the ash content might be associated with the loss of organic matter (Ismail et al. 2024). The particular increase in protein contents during fermentation could be linked to the biological activities of lactic acid bacteria, which hydrolyze proteins and peptides in food matrices in order to increase the amount of free amino acids and subsequently utilize them. LAB also produce secondary metabolites, including exopolysaccharides, enzymes, and bacteriocins, which are used to increase the nutritional quality, shelf‐life of fermented foods, and health benefits (Falah et al. 2019; Tachie et al. 2024). Dietary fiber increase is considered good in promoting good health, such as digestive and gut health (Alahmari 2024). Our results for the nutritional composition are comparable with earlier studies by Demirgül and Ozturk (2021), Irakoze et al. (2023), and Simões et al. (2022), wherein the researchers elucidated the highest contents of ash, proteins, and fibers in fermented tomato powder, that is, 2–13, 4–41, and 2–23 g/100 g, respectively. However, another study by Shakouri et al. (2023) portrayed a significant (*p* < 0.05) increase in ash and proteins by 16.5% and 25% in fermented tomato pomace powders, which is closely aligned with our research results.

### 3.2 Mineral Composition of Blanched, Fermented and Ultrasound‐Treated Tomato Powder

Among the results of inorganic elements, the untreated and treated versions of tomato powders anticipated the highest K, Na, Mg, Ca, Fe, and Zn contents, that is, 2246, 792, 271, 158, 5.57, and 1.98 mg/100 g, respectively, in the LAB fermentation, followed by the lowest in untreated tomato powder, that is, 1822, 537, 194, 143, 4.88, and 1.78 mg/100 g, respectively. Following fermentation, ultrasonicated (i.e., 20 min) tomato powder exhibited higher K, Na, Mg, Ca, Fe, and Zn, that is, 1934, 651, 234, 151, 5.24, and 1.88 mg/100 g, respectively. The blanched tomatoes also exhibited significant (*p* < 0.05) increments in K, Na, Mg, Ca, Fe, and Zn in tomato samples, that is, 1902, 590, 221, 150, and 5.1 mg/100 g, respectively, which indicates health benefits of tomatoes, which could be health benefiting for humans (Table 1). LAB fermentation improved the inorganic micronutrients, overall quality, and bioavailability, owing to the release of bound minerals from the antinutrients (Barzegar et al. 2023; Rollán et al. 2019). The highest levels of mineral elements in the fermented tomato powder samples could directly correlate with the hydrolysis of insoluble mineral‐antinutrient complexes such as phytates and tannins (Knez et al. 2023). Dietary intake of essential inorganic residues is considered an essential indicator of minerals' quality, which is viable in maintaining good human health and impactful in progressing biochemical processes (Razzaque and Wimalawansa 2025). Minerals such as Ca, Na, and K support performing muscle functions, blood clotting, maintaining blood pressure, and regulating electrolytes (Yadav et al. 2024). Magnesium supports metabolism and cell growth. Iron and zinc support oxygen transport, immunity, cellular growth, and enzymatic regularities (Fatima et al. 2024). Comparable findings for minerals in tomato powder have also been revealed in an earlier study by Simões et al. (2022), wherein the authors showed the highest minerals in fermented tomato pulp samples, that is, K, Na, Mg, Ca, Fe, and Zn, that is, 2254, 885, 260, 157, 5.7, and 2.4 mg/100 g, respectively. Another retrospective study by Ajayi and Awe (2022) demonstrated the maximum concentrations of K, Na, Mg, and Ca in the fermented *Solanum aethiopicum* powder, that is, 184, 346, 194, and 207 mg/100 g, respectively.

**TABLE 1:** Mineral composition of blanched, fermented, and ultrasound‐treated tomato powder (mg/100 g).

Treatments	Na	K	Ca	Mg	Fe	Zn
UT	537 ± 2.82^f^	1822.5 ± 1.53^f^	143.85 ± 0.82^f^	194.38 ± 1.1^f^	4.88 ± 0.23^e^	1.78 ± 0.05^e^
FT24	792.5 ± 3.53^a^	2246.5 ± 1.12^a^	158.89 ± 0.79^a^	271.4 ± 1.05^a^	5.56 ± 0.35^a^	1.97 ± 0.07^a^
BL	590.15 ± 2.15^c^	1902 ± 1.06^c^	150.18 ± 0.57^c^	221.33 ± 1.13^c^	5.07 ± 0.21^d^	1.83 ± 0.09^c^
US10	553.26 ± 1.07^e^	553.26 ± 2.72^e^	145.47 ± 0.82^e^	198.31 ± 1.04^e^	4.92 ± 0.31^e^	1.82 ± 0.06^d^
US20	651.6 ± 2.16^b^	1934.47 ± 1.06^b^	151.78 ± 0.74^b^	234.56 ± 0.90^b^	5.24 ± 0.33^b^	1.88 ± 0.08^b^
US30	578.35 ± 1.12^d^	1865.4 ± 1.08^d^	147.37 ± 0.82^d^	215.35 ± 0.87^d^	5.11 ± 0.22^c^	1.86 ± 0.09^bc^

*Note:* Values are expressed as means ± SD (*n = 2*). Mean values presenting similar lettering in a column are statistically nonsignificant (*p* > 0.05).

Abbreviations: BL, blanching; FT24, fermentation 24 h; US10, ultra‐sonication 10 min; US20, ultra‐sonication 20 min; US30, ultra‐sonication 30 min; UT, untreated tomatoes.

### 3.3 Functional Attributes of Blanched, Fermented and Ultrasound‐Treated Tomato Powder

#### 3.3.1 Chlorophyll Content

Among the results for a color profile of tomato powders, untreated tomato powder exhibited (*p* < 0.05) higher chlorophyll *a*, chlorophyll *b*, and total chlorophyll, that is, 11.6, 7.8, and 19.40 mg/100 g significantly, respectively. However, among treatments, the fermented, blanched, and ultrasonicated tomato powders portrayed slightly lower magnitudes of chlorophyll *a*, chlorophyll *b*, and total chlorophyll, which ranged between 4.13–9.3, 3.2–6.1, and 7.4 and 15.4 mg/100 g, respectively (Table 2). The lowest losses of chlorophyll *a*, chlorophyll *b*, and total chlorophyll were recorded in the tomato powders ultrasonicated at 20 min, that is, 9.3, 6.1, and 15.46 mg/100 g, which could be associated with the swelling and hydration ability of coloring pigments that ultimately enlarge the cell wall pores and retain the chlorophyll (Manzoor et al. 2021). Chlorophyll is known to be pH sensitive; during fermentation, lactic acid production decreases pH, which subsequently contributes to chlorophyll degradation (Al‐Obaidi and Alsawaf 2024; Degrain et al. 2020). The chlorophyll losses during blanching are likely attributed to high‐temperature exposure (Nguyen et al. 2023). The presence of dietary chlorophyll indicates its antioxidant activities, which are helpful in alleviating a number of health disorders such as cancer (Rueangsri et al. 2025; Yang et al. 2024). Comparable findings for total chlorophyll were reported by Sangija et al. (2022), wherein the authors portrayed the fermentation to significantly reduce the total chlorophyll from 392.5 to 164.5 mg/100 g in African nightshade. Likewise, another study corroborates our findings, wherein the researchers reported a similar decline in chlorophyll *a* and *b* from 139 to 16.6 mg/100 g in the fermented green bell pepper, respectively.

**TABLE 2:** Functional attributes of blanched, fermented, and ultrasound‐treated tomato powder.

Treatments	Chlorophyll *a*	Chlorophyll *b*	Total chlorophyll	Lycopene (mg/100 g)	β‐carotene (mg/100 g)	Ascorbic acid (mg/100 g)
UT	11.57 ± 0.09^a^	7.82 ± 0.16^a^	19.40 ± 0.12^a^	137.37 ± 1.21^f^	8.25 ± 0.21^f^	121.35 ± 1.24^a^
FT24	7.27 ± 0.12^c^	5.82 ± 0.13^b^	13.09 ± 0.17^c^	176.95 ± 1.24^a^	14.76 ± 0.12^a^	113.25 ± 1.39^b^
BL	4.13 ± 0.13^e^	3.28 ± 0.09^c^	7.41 ± 0.14^e^	156 ± 1.17^c^	6.25 ± 0.19^d^	53.33 ± 1.22^f^
US10	4.12 ± 0.07^e^	3.46 ± 0.08^c^	7.58 ± 0.11^e^	143.68 ± 1.31^e^	8.96 ± 0.27^e^	78.96 ± 1.72^e^
US20	9.31 ± 0.16^b^	6.15 ± 0.09^b^	15.46 ± 0.19^b^	158.36 ± 1.13^b^	10.25 ± 0.20^b^	93.17 ± 1.17^c^
US30	5.36 ± 0.15^d^	4.25 ± 0.12^c^	9.60 ± 0.14^d^	153.28 ± 1.12^d^	9.46 ± 0.18^c^	81.62 ± 0.58^d^

*Note:* Values are expressed as means ± SD (*n = 2*). Mean values presenting similar lettering in a column are statistically nonsignificant (*p* > 0.05).

Abbreviations: BL, blanching; FT24, fermentation 24 h; US10, ultra‐sonication 10 min; US20, ultra‐sonication 20 min; US30, ultra‐sonication 30 min; UT, untreated tomatoes.

#### 3.3.2 β‐Carotenoid and Lycopene Contents

Among lycopene and beta‐carotene contents of untreated and treated versions of tomato powders, the maximum concentrations were observed in the LAB fermented tomato powders, that is, 177 and 15 mg/100 g, followed by the lowest mean concentrations of these pigments in untreated tomato powder, that is, 137 and 8.3 mg/100 g, respectively. Following fermentation, tomato powders ultrasonicated at 20 min showed the highest lycopene and beta‐carotene contents, that is, 158 and 10.3 mg/100 g, respectively (Table 2). However, blanching of tomato powder delineated a significant (*p* < 0.05) decline in beta‐carotene from 8.3 to 6.2 mg/100 g, which could be in connection with the thermal breakability, leaching, and percolation losses (Sarkar et al. 2021). Beta‐carotene is known for its health significance such as it is known to prevent cardiovascular disorders, breast cancer, prostate cancer, lung cancer, colon cancer, diabetes, and obesity and promotes skin health (Ebadi et al. 2023). Lycopene exhibits significant contribution to human health against atherosclerosis, endothelial dysfunction, inflammation, and high blood pressure (Khan et al. 2021).

However, increased lycopene contents in the fermented tomato powder could be associated with activities of exogenous enzymes and disruption of cellular walls, subsequently releasing bound lycopene (Kaur and Ghosh 2023). Beta‐carotene improvement during fermentation might also be correlated with the bacterial enzymatic activities that enhance the carotenoids (Mapelli‐Brahm et al. 2020). Beta‐carotene and carotenoids are natural colorants with viable antioxidant properties and a number of health benefits which portray their role in mitigating a number of health disorders such as cancer, diabetes, and blood pressure (Sharma et al. 2024). Similar to our results, a group of researchers consisting of Mechmeche et al. (2022) elucidated significant (*p* < 0.05) higher lycopene contents in the LAB fermented tomato slices, that is, 23.8 mg/100 g. Also, Sangija et al. (2022) delineated significantly (*p* < 0.05) higher beta‐carotene concentrations in the fermented *Solanum scabrum* and *Solanum villosum* powders, that is, 48.7 and 31 mg/100 g, respectively. Another retrospective study by Etu et al. (2024) reported the highest beta‐carotene concentrations of 15 mg/100 g in the fermented eggplant leaves.

#### 3.3.3 Ascorbic Acid Content

Fermented, blanched, and ultrasonicated (i.e., 10, 20, and 30 min) tomato powders indicated significantly (*p* < 0.05) lower concentrations of ascorbic acid, i.e., 113, 53, and 79 mg/100 g, respectively, when compared with the control, which exhibited the higher magnitudes of ascorbic acid, that is, 121 mg/100 g (Table 2). Among the fermentation treatments, ultrasonication (10, 20, and 30 min) and blanching, the ascorbic acid contents varied between 53 and 113 mg/100 g. A notable decline in ascorbic acid in treated tomato powders could be co‐related to pH and thermal sensitivity, breakability, and higher water dissolution on blanching, fermentation, and ultrasonication (Sarkar et al. 2021). Ascorbic acid is a naturally occurring antioxidant that is involved in protecting the health and immune system in humans and also foliates an array of health functions, for example, decreases allergic responses and supports in fighting against infections and other cancers (Ali et al. 2024). Our findings are consistent with earlier studies by Ndungutse et al. (2024) and Saini et al. (2023), wherein the researchers elucidated a significant (*p* < 0.05) decline in mean concentrations of ascorbic acid from 12 to 1.3 and 56 to 37 mg/100 g in the fermented potatoes of the Solanaceae family. Another study by Sunmonu et al. (2021) delineated the blanching to decline the ascorbic acid contents from 38.8 to 29.6 mg/100 g in dehydrated tomato powder.

### 3.4 Antinutrient Contents of Blanched, Fermented and Ultrasound‐Treated Tomato Powder

Results for antinutrients of untreated and treated tomato powders revealed the highest contents of phytates, oxalate, glycoalkaloids, and saponins in untreated tomato powder, that is, 281, 298, 51, and 10.2 mg/100 g, respectively. Fermentation, blanching, and ultrasonication treatments caused a significant (*p* < 0.05) decline in phytates (88%–96%), oxalate (87%–94%), glycoalkaloids (49%–87%) and saponins (55%–89%). LAB‐fermented tomatoes demonstrated the highest %reduction in the phytates, oxalates, glycoalkaloids, and saponins by 95%, 94%, 87%, and 89%, respectively (Table 3).

**TABLE 3:** Antinutrient contents of blanched, fermented, and ultrasound‐treated tomato powder (mg/100 g).

Antinutrients	UT	FT24		BL		US10		US20		US30	
		Treatment	%R	Treatment	%R	Treatment	%R	Treatment	%R	Treatment	%R
Phytates	280.54 ± 2.27^a^	11.8 ± 0.52^f^	95.8	28.3 ± 0.14^d^	89.9	40.3 ± 0.53^b^	85.6	25.9 ± 0.37^e^	90.7	32.23 ± 0.19^c^	88
Oxalates	298.4 ± 2.32^a^	18.21 ± 0.16^f^	94.3	30.35 ± 0.11^d^	89	36.24 ± 0.21^b^	87	26.76 ± 0.51^e^	91	32.81 ± 0.27^c^	89
Glycoalkaloids	50.77 ± 1.01^a^	6.43 ± 0.08^f^	87	17.46 ± 0.16^d^	65.5	25.89 ± 0.17^b^	49	11.16 ± 0.47^e^	78	21.19 ± 0.22^c^	58
Saponins	10.24 ± 1.24^a^	1.22 ± 0.03^f^	89	3.9 ± 0.14^d^	61	4.54 ± 0.08^b^	55	3.2 ± 0.09^e^	69	4.12 ± 0.11^c^	60

*Note:* Values are expressed as means ± SD (*n = 2*). Mean values presenting similar lettering in a column are statistically nonsignificant (*p* > 0.05).

Abbreviations: %R, % reduction; BL, blanching; FT24, fermentation 24 h; US10, ultra‐sonication 10 min; US20, ultra‐sonication 20 min; US30, ultra‐sonication 30 min; UT, untreated tomatoes.

Among antinutrients, the oxalates interfere with the Ca^2+^ and result in affecting the absorption and reducing its bioavailability owing to the formation of calcium oxalate salts (Wdowiak et al. 2024). The decline in contents of phytates, oxalates, saponins, and alkaloids in biological, thermal, and non‐thermal treatments could be linked with thermal breakability, cell destruction, dissolution, leaching losses, molecular disruption, and bond degradation (Arjmand et al. 2023; Manzoor et al. 2021). The reduction in phytates might be linked with the release of lactic acid, which significantly lowers the pH and subsequently activates phytase (Ayub et al. 2021). Oxalate and saponin reduction could also be associated with the secretion of β‐glucosidases, which cause leaching and hydrolysis (Sulaiman et al. 2020). Our findings for the antinutrients of tomato powders are in line with an earlier study by Okhonlaye et al. (2020), wherein the researchers elucidated a noticeable decline in phytates and oxalates from 10.7 to 5.6 and 25 to 13.6 mg/100 g in Irish potato peels, respectively. Likewise, another finding by Minh (2022) reported a positive correlation of fermentation in declining the phytates and glycoalkaloids in African eggplant from 127 to 9.4 and 49.6 to 0.8 mg/100 g, respectively. In addition, Sangija et al. (2022) delineated that the fermentation of African nightshade resulted in a 77%–90% reduction in oxalates. Another retrospective by Gong et al. (2024) anticipated a significant (*p* < 0.05) effect of fermentation in decreasing the glycoalkaloids from 14 to 11 mg/100 g in potato flour. Also, Ajayi and Awe (2022) depicted the fermentation to alleviate saponins from 11 to 10 mg/100 g of Garden eggplant.

### 3.5 Antioxidant Activities of Blanched, Fermented and Ultrasound‐Treated Tomato Powder

#### 3.5.1 Total Phenolic Content (TPC)

Results for total phenolics of untreated and treated tomato powders demonstrated the maximum TPC in the LAB fermented tomato powder, that is, 765 mg GAE/100 g, followed by the lowest in control, that is, 655 mg GAE/100 g, respectively. However, among the treatment groups, ultrasonication (i.e., 20 min) and blanching exhibited moderately higher TPC, that is, 687 and 681 mg GAE/100 g, respectively (Table 4). A notable increase in antioxidant activities of LAB in the fermented products could be in close harmony with the proteolytic actions and phenolics production, which results in the development of antioxidants that ultimately exhibit higher antioxidant activities (Mousanejadi et al. 2023; Sarıtaş et al. 2024; Yazdi et al. 2019). Polyphenols played health‐featuring role in eliminating reactive free oxygen species, which are instigators of a number of health illnesses, which also help in improving the essential lipid profiles, maintaining the blood pressure, insulin regulations, and systemic anti‐inflammatory properties (Aljohani and Zaman 2024; Behbahani, Jooyandeh, Hojjati, et al. 2024; Rudrapal et al. 2024).

**TABLE 4:** Antioxidant activities of blanched, fermented, and ultrasound‐treated tomato powder.

Treatments	TPC (mg GAE/100 g)	TFC (mg QE/100 g)	DPPH %	FRAP (μmol Fe^+2^/100 g)
UT	655.39 ± 3.39^f^	537.66 ± 1.27^f^	70 ± 1.19^c^	37.1 ± 0.89^f^
FT24	764.66 ± 2.29^a^	647.30 ± 1.52^a^	87.47 ± 1.03^a^	56.35 ± 1.22^a^
BL	681.34 ± 2.43^c^	573.36 ± 1.39^c^	73.33 ± 1.80^c^	44.56 ± 1.01^c^
US10	661.77 ± 2.77^e^	542.47 ± 2.32^e^	71.30 ± 1.21^d^	41.38 ± 1.09^e^
US20	687.98 ± 3.51^b^	583.68 ± 1.41^b^	76.55 ± 1.29^b^	46.60 ± 1.33^b^
US30	671.80 ± 3.33^d^	559.74 ± 2.29^d^	67.34 ± 1.25^f^	42.46 ± 1.41^d^

*Note:* Values are expressed as means ± SD (*n = 2*). Mean values presenting similar lettering in a column are statistically nonsignificant (*p* > 0.05).

Abbreviations: BL, blanching; FT24, fermentation 24 h; US10, ultra‐sonication 10 min; US20, ultra‐sonication 20 min; US30, ultra‐sonication 30 min; UT, untreated tomatoes.

The increase in total phenolics during fermentation is due to lactic acid bacteria hydrolyzing macromolecules, releasing the bound phenolics, and LAB‐produced organic acids may also increase phenolics bio‐availabilities (Knez et al. 2023). LAB release enzymes, which tend to increase the biotransformation of phenolics and subsequently enhance antioxidant activities (Alam 2021; Rouhi et al. 2024; Zibaei‐Rad et al. 2023). The increase in TPC during ultrasonication could be correlated with the disruption of cellular walls, which results in a release of bound phenolics (Manzoor et al. 2023). Earlier research by Irakoze et al. (2023) reported a comparable increase in TPC in fermented African black nightshade from 21,840 to 22,880 mg GAE/100 g. Likewise, another study by (Degrain et al. 2020) reported a significant (*p* < 0.05) increase in TPC from 600 to 863 mg GAE/100 g in the fermented leaves of the nightshade plant. Also, Nzimande et al. (2024) revealed a significant increase in TPC from 74.8 to 87.3 mg/100 g on ultrasonication of tomatoes. The increment of TPC during ultrasonication could be attributed to the ability of ultrasound cavitation to enhance the extraction of bound phenolics by breaking the plant cell wall (Akcicek et al. 2023). Further, cavitation‐induced hydroxylation of phenolic rings could have been linked to release‐bound phenolics (Manzoor et al. 2023). Furthermore, da Costa et al. (2023) showed an increment in phenolics from 33.6 to 56.8 mg/100 g on thermal processing of tomato pomace.

#### 3.5.2 Total Flavonoid Content (TFC)

Among untreated and treated tomato powders, the highest TFC was reported in fermented tomato powder, that is, 647 mg/100 g, compared to the control, that is, 537 mg/100 g. However, among treatments, the ultrasonication (i.e., 20 min) and blanching portrayed slightly lower magnitudes of TFC, that is, 583 and 573 mg/100 g, respectively (Table 4). Flavonoids are natural compounds which act as antioxidants and are involved in showing great protective effects against some chronic disorders like neurodegenerative diseases, cardiovascular diseases, and cancer (Yang et al. 2023). The increase in TFC in fermented tomato powder could be directly linked with the secretion of endogenous enzymes that are known to hydrolyze the covalent bonds, thereby dissociating glycosidic bonds in phenolics and flavonoids and resulting in the release of flavonoid aglycones (Du et al. 2024). Our findings are comparable with the earlier research studies conducted by Minh (2022), wherein the researchers reported a significant (*p* < 0.05) increase in the TFC of fermented eggplant from 2339 to 3620 mg/100 g, respectively. Likewise, another finding by Irakoze et al. (2023) elaborated that fermentation significantly increases the TFC from 667 to 1096 mg/100 g in African black nightshade. Comparable findings for total flavonoid content were also reported by da Costa et al. (2023), wherein heat treatments significantly increased flavonoid contents in tomato peel from 58.1 to 77.5 mg/100 g (i.e., 33.55% increase).

#### 3.5.3 DPPH Antioxidant Activity

DPPH results exhibited the highest activities in the fermented tomato powder, that is, 87.5%, followed by ultrasonication (i.e., 20 min), blanching, ultrasonication (i.e., 10 min), and ultrasonication (i.e., 30 min), that is, 76.5%, 73.3%, 71.30%, and 67.34%, respectively. The increase in tomato powder's free radical scavenging antioxidant activity during the fermentation process could be associated with the LAB‐tannases and feruloyl esterases, which cause a release of bound phenolics, ultimately enhancing the DPPH activities (Table 4). An earlier study by Mechmeche et al. (2022) observed notable DPPH activities in LAB‐fermented tomato powder, that is, 33%. Similarly, our findings align with those of Liu et al. (2024), wherein the researchers revealed appreciable DPPH activities, that is, 67% in the Solanaceae family's LAB‐fermented wolfberry pulp. Also, Hussain et al. (2021) exhibited significant enhancement in DPPH activities of tomatoes from 21% to 51% in pretreated potatoes of the Solanaceae family. Earlier, in a study by Zhou et al. (2022), a notable increase in DPPH activities of tomatoes was recorded at 16% in hot air blanching of tomatoes compared to fresh tomatoes.

#### 3.5.4 Ferric Reducing Antioxidant Power (FRAP)

Among findings for the FRAP contents of untreated and treated versions of tomato powders, the highest of its contents was observed in the LAB fermented tomato powder, that is, 56.4 μmol Fe^+2^/100 g, followed by the lowest mean concentrations of FRAP in control, that is, 37 μmol Fe^+2^/100 g. Among treatments, blanched and ultrasonicated (10, 20, and 30 min) tomato powders revealed relatively lower FRAP concentrations, that is, 44.6, 41, 47, and 42 μmol Fe^+2^/100 g, respectively (Table 4). The increment in FRAP activities of tomato powder during fermentation is attributed to the release of polyphenols by enzymatic activities of LAB (Degrain et al. 2020). Comparable findings for the FRAP contents were also reported by Etu et al. (2024), wherein the authors showed a significant (*p* < 0.05) increment in FRAP mean values from 68.6 to 74 μmol Fe^+2^/100 g in fermented garden egg leaves. Likewise, Selimović et al. (2023) anticipated a significant (*p* < 0.05) increase in FRAP contents of tomatoes from 4401 **to** 4869 μmol Fe/100 g at 50°C–75°C.

### 3.6 pH, Titratable Acidity, and Microbial Counts

**FIGURE 2:**
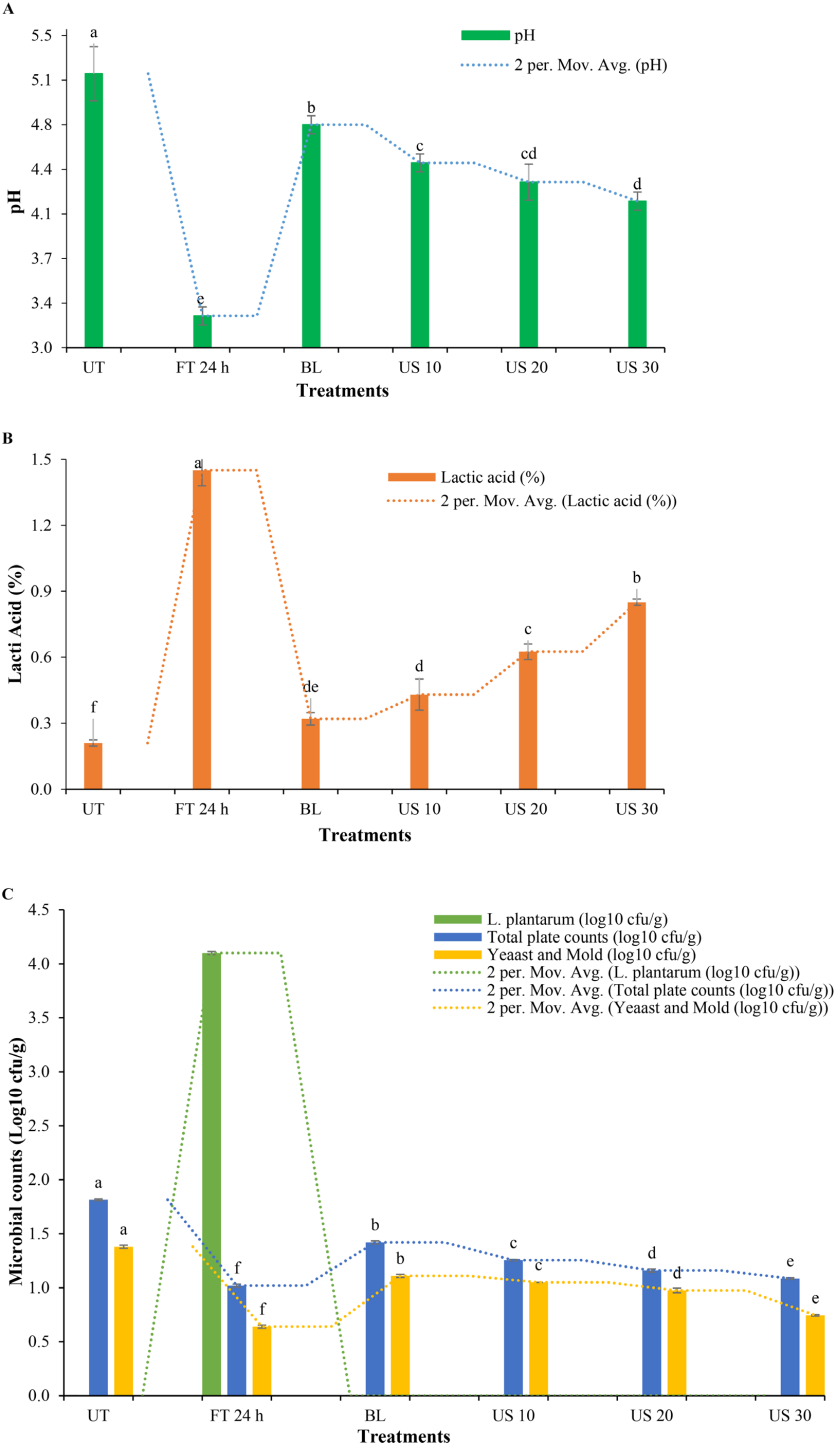
(A) pH, (B) lactic acid (%), and (C) microbial ( *L. plantarum* , total plate, yeast and mold) counts of untreated tomatoes (UT), fermentation 24 h (FT24), blanching (BL), ultrasonication 10 min (US10), ultrasonication 20 min (US20), and ultrasonication 30 min (US30). Values are expressed as means ± SD (*n* = 2). Mean values presenting similar lettering are statistically nonsignificant (*p* > 0.05).

Our findings for the pH and titratable acidity (i.e., lactic acid %) of untreated, fermented, blanched, and ultrasonicated tomato samples showed a significant decline in pH and increased titratable acidity as the fermentation proceeds. The pH value varied between 5.15 and 3.25 on fermentation at 0–24 h of LAB fermentation (Figure 2A). A sharp drop of pH during the fermentation could be associated with the activities of lactic acid bacteria which produced a significant amount of acid during their metabolisms (Stoll et al. 2021). Also, the increase in titratable acidity of fermented tomatoes could be co‐related to the metabolization of sugars into lactic acid, which releases H^+^ ions (Karow et al. 2024). Thus, LAB fermentation is a desirable biological activity that maintains highly acidic conditions. Our findings are comparable with Ndungutse and Njiraine (2022), who reported a significant (*p* < 0.05) decline in the pH of fermented *Solanum tuberosum* from 7.3 to 4.3. The titratable acidity of the LAB fermented tomato powder measured as lactic acid (%) was noticed to be increased from 0.21% to 1.45% after 24 h of fermentation (Figure 2B). Similarly, another retrospective study by Sangija et al. (2022) delineated a significant (*p* < 0.05) decline in the pH of LAB‐fermented African nightshade from 7.4 to 3.5 with a titratable acidity range of 0.045%–0.4%.

Results for the *L. plantarum* counts of tomatoes fermented at 0 and 24 h revealed a significant (*p* < 0.05) increase in the *L. plantarum* counts from 0 to 4.11 log_10_ CFU/g, respectively (Figure 2C). In comparison to our study, comparable findings for the *L. plantarum* counts were also reported by Mocanu et al. (2022) wherein the *L. plantarum* log_10_ CFU/g in fermented tomatoes was increased from 7 to 7.5 log_10_ CFU/g. In another study by Mechmeche et al. (2022), the researchers portrayed a significant increase in lactic acid bacteria counts from 4.11 to 4.63 log_10_ CFU/g in fermented tomatoes. *L. plantarum* orients a number of health benefits as a probiotic against gastrointestinal infections, inflammation, bowel diseases, and stimulating effects on the immune system (Zibaei‐Rad et al. 2024). Moreover, this probiotic can also prevent human health against pathogenesis and increase the shelf life in bio‐processed foods through its natural antimicrobial abilities (Behbahani, Jooyandeh, Taki, et al. 2024; Hojjati et al. 2020; Zare et al. 2024). The increase in the *L. plantarum* count during fermentation could be correlated to the availability of sugars, which are viable carriers of bacterial growth and reproduction (Mocanu et al. 2022). Likewise, Pereira et al. (2023) also demonstrated a significant increase in the lactic acid bacterial counts in fermented tomatoes from 7.5 to 9 log_10_ CFU/mL.

Findings for total plate counts of fermented (0 and 24 h) tomatoes revealed a significant (*p* < 0.05) decrease in the total plate counts from 1.81 to 1.02 log_10_ CFU/g, respectively (Figure 2C). Perveen et al. (2024) showed a significant reduction in the total plate counts of fermented *M. oleifera* leaves from 1.75 to 1.24 log_10_ CFU/g. Total mold count results of fermented (0 and 24 h) tomatoes revealed a significant (*p* < 0.05) decrease in the mold counts from 1.38 to 0.64 log_10_ CFU/g, respectively (Figure 2C). Similarly, Ndungutse and Njiraine (2022) portrayed the significant decline in mold counts of potato cultivars from 4.91 to 4.4 log_10_ CFU/g of the Solanaceae family. Also, Perveen et al. (2024) reported that lactic acid fermentation reduced the mold's counts from 1.41 to 0.92 log_10_ CFU/g in the fermented *M. oleifera* leaves.

## 4 Conclusions

The key findings of this investigation suggest tomato powder is a viable source of health‐promoting nutrients such as ash, fiber, high‐quality proteins, lycopene, beta‐carotene, and essential micronutrients, which are known to contribute to health‐ameliorating features on consumption. Antagonistically, higher magnitudes of toxicogenic antinutrient compounds in tomato powder above permissible limits result in chronic to acute health ailments and micronutrient inadequacies. Among the results, *L. plantarum* and titratable acidity of fermented tomatoes were increased; however, total plate and mold counts were significantly decreased. The present investigation exhibited all the processing techniques, including blanching, ultrasonication, and lactic acid fermentation, to improve ash, fiber, and phenolics and reduce loads of oxalates, phytates, saponins, and glycoalkaloids in tomato powder. Conclusively, among all thermal and non‐thermal processing techniques, LAB fermentation (i.e., 24 h) exhibited the highest reduction of antinutrients by 89%–96%, contributing to the safety and nutrient improvement in tomato powder. In the backdrop of the present study, further studies are required to evaluate the toxicological impacts of treated tomato powder in animal modeling and its utilization as a valuable ingredient in sauces, curries, gravies, and soups.

## Author Contributions

**Abdul Rehman Ayub:** conceptualization (equal), formal analysis (equal), software (equal), supervision (equal), writing – original draft (equal). **Muhammad Waseem:** conceptualization (equal), investigation (equal), project administration (equal), writing – review and editing (equal). **Zulfiqar Ahmad:** data curation (equal), software (equal), writing – original draft (equal), writing – review and editing (equal). **Jaza Maqbl Alshammari:** conceptualization (equal), investigation (equal), supervision (equal), writing – review and editing (equal). **Tariq Ismail:** conceptualization (equal), investigation (equal), software (equal), writing – review and editing (equal). **Muhammad Ammar Khan:** conceptualization (equal), data curation (equal), methodology (equal), software (equal), writing – original draft (equal). **Muhammad Rizwan Javed:** conceptualization (equal), data curation (equal), formal analysis (equal), software (equal), writing – original draft (equal). **Muhammad Saleem:** formal analysis (equal), project administration (equal), supervision (equal), writing – review and editing (equal). **Rana Muhammad Aadil:** data curation (equal), formal analysis (equal), investigation (equal), writing – review and editing (equal). **Muhammad Faisal Manzoor:** formal analysis (equal), project administration (equal), supervision (equal), writing – original draft (equal). **Muhammed Adem Abdullahi:** project administration (equal), supervision (equal), writing – review and editing (equal).

## Conflicts of Interest

The authors declare no conflicts of interest.

## Data Availability

The datasets in this study are available from the corresponding author on reasonable request.
